# Tackling Dyslipidemia in Obesity from a Nanotechnology Perspective

**DOI:** 10.3390/nu14183774

**Published:** 2022-09-13

**Authors:** Laura M. Trandafir, Gianina Dodi, Otilia Frasinariu, Alina C. Luca, Lacramioara I. Butnariu, Elena Tarca, Stefana M. Moisa

**Affiliations:** 1 Pediatrics Department, Grigore T. Popa University of Medicine and Pharmacy of Iasi, 700115 Iasi, Romania; 2 Advanced Research and Development Center for Experimental Medicine, Grigore T. Popa University of Medicine and Pharmacy of Iasi, 700454 Iasi, Romania; 3 Department of Medical Genetics, Grigore T. Popa University of Medicine and Pharmacy of Iasi, 700115 Iasi, Romania; 4 Department of Pediatric Surgery, Grigore T. Popa University of Medicine and Pharmacy of Iasi, 700115 Iasi, Romania

**Keywords:** dyslipidemia, obesity, nanotechnology, drug delivery, natural products

## Abstract

Obesity and dyslipidemia are the main features of metabolic syndrome, expressed mainly by adipose tissue dysfunction and connected by similar pathways and pharmacotherapy. Conventional drugs used in these two associated disorders are limited due to poor drug efficiency, non-specificity, and toxic side effects. Therefore, novel solutions for tackling obesity-associated diseases and providing insights into the development of innovative or improved therapies are necessary. Targeted nanotherapy is a revolutionary technology, offering a promising solution for combatting the disadvantages of currently available therapies for treating obesity and dyslipidemia due to its superior features, which include specific cell targeting, the protection of drugs against physiological degradation, and sustained drug release. This review presents a brief assessment of obesity and dyslipidemia, their impacts on human health, current treatment, and limitations, and the role and potential use of nanotechnology coupled with targeted drug delivery and nutraceuticals as emerging therapies. To the best of our knowledge, this paper presents, for the first time in the literature, a comparison between obesity and dyslipidemia nano-formulations based on drugs and/or natural extracts applied in experimental studies.

## 1. Introduction

Obesity is a health problem of worldwide interest with negative impacts on quality of life [1], being a chronic and multifactorial disease, as defined by the Disease International Classification [2]. The World Health Organization (WHO) defines overweight and obesity as the abnormal collection of fat inside the body, leading to health-related consequences. The most frequently used method of measuring and identifying obesity is the body mass index (BMI). In adults, obesity corresponds to BMI ≥ 30 kg/m^2^, but in children the definition criteria also include age. Thus, in children younger than 5, obesity is defined as a weight-to-height ratio more than three standard deviations over the WHO child growth standard median, and for children aged 5 to 19, obesity is defined as a weight-to-height ratio more than 2 standard deviations over the WHO child growth standard median [3]. Individually, the BMI remains an important screening tool that is quick and easy to use, and it is considered appropriate for monitoring worldwide disease prevalence and quantifying epidemiological studies [4]. Along with BMI, the waist circumference (WC) represents another index of central obesity. This is a clinical parameter that determines the nutritional status independent of BMI [5]. However, even if both BMI and WC are considered indices that provide independent information about the outcomes of patients, there are also limitations to their use, such as in patients with coronary artery disease [6]. The recommended indication suggests that the assessment of obesity should be performed on patients prior to revascularization interventions for a more accurate prognosis. According to the clinical perspective published by Han and Lean [7], BMI is not a strong predictor of body fat, mainly because the degree of variance in body fat is 70–80% in adults. Therefore, a more rational approach is to use a combination of anthropometric measurements in order to estimate body fat [8].

The prevalence of obesity has increased worldwide over recent years and has now reached pandemic levels [9]. At least 2.8 million people die every year as a consequence of obesity, which supports the pandemic hypothesis [1]. By 2030, it is estimated that one in five women and one in seven men will be living with obesity, meaning that there will be over 1 billion people battling obesity worldwide. In 2019, over 160 million healthy life years were lost, approximating more than 20% of healthy life years lost due to a non-communicable, preventable disease, and this number is probably going to become higher every year [4]. Obesity usually starts during childhood; therefore, infantile obesity represents an alarming public health issue. It was estimated that, in 2020, 39 million children under the age of 5 years were overweight or obese [3]. Heavy children have a 50% probability of becoming obese adults and developing typical pathologies, such as diabetes mellitus type-2 (T2DM), dyslipidemia, arterial hypertension, or psychosocial and emotional problems. Therefore, early prevention becomes an essential element, along with the early identification of infantile obesity risk factors that could greatly help physicians to establish adequate and specific treatments [10].

This review investigates the literature concerning the underlying nanotechnological assessment of dyslipidemia-related obesity based on a search of the PubMed engine using the following keywords: dyslipidemia, obesity, nanomedicine. Subsequently, studies from the last 15 years were screened, and relevant citations were included. This review aims to gather all the in vitro and in vivo studies regarding drug/plant-based nano-formulations of obesity and dyslipidemia.

## 2. Obesity and Its Interrelated Conditions

Obesity, like all chronic diseases, is associated with a wide range of determinants, including genetic and biological factors, medical assistance access, mental health, socio-cultural factors, over processed food, the economy, commercial and environmental elements [4], and neuronal and hormonal mechanisms [11]. All these represent the roots of obesity, interacting with and comprising a number of systems leading to the tendencies that we are witnessing today. In many societies, obesity is seen as a personal failure, and there is limited recognition of the wide range of determinants of obesity.

Obesity is associated with metabolic syndrome and T2DM, conditions that share a number of pathophysiological mechanisms that lead, in succession, to cardiovascular complications. Metabolic syndrome is characterized by abdominal adiposity, insulin resistance, dyslipidemia, endothelial dysfunction, and inflammation or thrombosis, being intensified by genetic conditioning, age, physical inactivity, and the consumption of high-calorie, high-fat foods, concentrated carbohydrates, and salt [12]. It was observed that, although obesity significantly increases the risk of developing metabolic disorders and cardiovascular diseases, there is a category of obese patients (up to 30%), defined as having metabolically healthy obesity (MHO), with no metabolic abnormalities despite their excessive body weight. These individuals have insulin sensitivity similar to that of healthy normal-weight individuals, a reduced visceral fat content, and lower intima media thickness of the carotid artery compared to the majority of individuals diagnosed with metabolically unhealthy obesity (MUO) [13]. Research data from 2021 showed that MHO carries a greater risk of developing cardiovascular diseases and cardiovascular events than that of normal-weight individuals but less than that of MUO patients, confirming that obesity is always a pathological condition, despite the lack of specific disease entities already developed [13]. In a 2022 study by Kruger et al. [14], this hypothesis was evaluated using the metabolic profiles of MHO and MUO South African adults over a 10-year period. The results showed that half of the MHO adults maintained their initial health state over the follow-up period, while the other half were no longer metabolically healthy by the end of the study. Regarding the MUO group, the results indicated that this state was associated with a higher fasting glucose and adverse lipid profile compared with the MHO group. Overall, the authors acknowledged that the MHO condition is transient and not in agreement with optimal health; thus, overweight and obese young adults should be included in health promotion programs [14].

Furthermore, obesity contributes to perivascular adipose tissue (PVAT) dysfunction, since it induces increased oxidative stress, an inflammatory state, and hypoxia. PVAT is located around the aorta, coronary arteries, small vessels, and vasculature of the musculoskeletal system as an additional special type of adipose tissue surrounding the blood vessels [15]. Although the exact mechanism of vascular dysfunction in obesity is still not well understood, there are some pathways, such as the increased activation of renin–angiotensin–aldosterone, PVAT-derived factor dysregulation, decreased bioavailability of nitric oxide (NO), and an increased inflammatory state in PVAT, which are involved in hypertension and endothelial dysfunction development. Physical activity and a healthy diet, commonly known as essential non-pharmacological intervention methods of obesity treatment, may have a positive impact on PVAT-derived factors, as well as on vascular tone and the re-establishment of the balance. However, additional studies should be conducted in order to improve our understanding of the pathophysiology of PVAT, to evaluate the influence of diet on PVAT among humans, and to assess whether targeting the PVAT function could be used as a novel approach for the treatment of cardiovascular diseases.

Subsequent to developing obesity as a health problem, people living with obesity face disgrace and suffer from impaired mental health. Therefore, obesity requires proper management and efficient treatment. Heavyweight people need adequate attention from healthcare professionals and multidisciplinary teams. Obesity is also relapsing, meaning that, without proper care, individuals remain exposed to the same related risks [4]: cardiovascular disease, diabetes, osteoarthritis, hepatic and renal disease, depression, sleep apnea [1], endometrial, breast, prostate, hepatic, gallbladder, renal, and colon cancer [3], as well as severe infection [16], stroke, Alzheimer disease, disability, and premature death [17,18].

Given that obesity is a chronic disease, its treatment should be integrated into the universal health systems as an essential health service in order to ensure equitable access and adequate care for all those facing this problem [4]. The main approaches used to tackle excess weight and obesity are diet modification, exercise, pharmacotherapy, and bariatric surgery [1].

Unfortunately, the promotion of healthy food habits and physical exercise is not always achievable, and obesity’s prevalence continues to intensify. Therefore, innovative solutions are required in order to promote non-obese food habits. Moreover, developing new pharmacological therapies to prevent and treat obesity are absolutely necessary [17].

## 3. Obesity and Dyslipidemia

Dyslipidemia is considered an obesity hallmark, posing serious risks to the affected patients’ health. Obesity and dyslipidemia are considered main players in metabolic syndrome due to the presence of adipose tissue dysfunction. Adipose tissue is an active endocrine (secrets adipokines) and immune organ inhabited by mature adipocytes, fibrous connective tissue, collagen, nerve cells, blood vessels, mesenchymal cells, fibroblasts, preadipocytes, endothelial precursor cells, smooth muscle cells, vascular cells, and macrophages [19]. Its main physiological roles are the regulation of food intake and the regulation of body weight, inflammation, coagulation, insulin sensitivity, or vascular function.

In obesity, hypertrophic adipocytes secrete high levels of pro-inflammatory adipokines and free fatty acids (FFA), leading to inflammation, dyslipidemia, and ectopic fat build-up. Additionally, increased plasma low-density lipoprotein (LDL) can be consumed by macrophages and introduced into the vascular sub-endothelium, which is implicated in the atherosclerosis cascade, suggesting that dyslipidemia is an established key factor of cardiovascular disease [20]. Studies have shown that the atherosclerosis prevalence rate increases with increased BMI, while patients using hypolipidemic therapy show improvements in symptoms of dyslipidemia and dyslipidemia-associated cardio-metabolic abnormality. Therefore, dyslipidemia has significant impacts on obesity and cardiovascular disease pathogenesis [21].

Several studies have shown the correlation between dyslipidemia and BMI [19]. The higher the BMI is, the more severe the lipid metabolism’s impairment is. Both obesity and overweight are associated with dyslipidemia: a total of 60–70% of obese patients and 50–60% of overweight patients are dyslipidemic [22].

The physiopathology of typical obesity-related dyslipidemia is multifactorial and includes several symptoms, as presented in Table 1.

It must also be highlighted that the effects of obesity on the lipid metabolism are also dependent on the adipose tissue localization. Visceral adipose layer and upper truncal subcutaneous tissue increase are associated with higher TG and lower HDL cholesterol levels. Moreover, visceral and upper truncal fat are associated with insulin resistance, which could contribute to the lipid abnormalities described above. Conversely, lower limb adipose tissue increase is associated with lower TG levels. The protective effects of lower limb fat could explain why women and African Americans have lower TG levels [25].

There are three major sources of fatty acids in the liver, all of which may be modified in obese patients. Firstly, the fatty acid flow to the liver is increased. Increased adipose tissue, especially the visceral deposits, result in an increased delivery of fatty acids to the liver. Furthermore, insulin suppresses TG lipolysis to produce FFA in the fatty tissue. In obese patients, decreased insulin activity due to insulin resistance leads to the inhibition of TG lipolysis and increased TG degradation in the adipose tissue, causing boosted fatty acid liver release. A second source of liver fatty acids is de novo synthesis. Numerous studies have shown that liver fatty acid synthesis is increased in obese patients. This increase can be mediated by the hyperinsulinemia, as noticed in insulin-resistant patients. More specifically, insulin stimulates the activity of sterol regulatory element-binding proteins-1c (a transcription factor that increases the necessary enzyme expression for fatty acid synthesis). A third source of fatty acids is the absorption of TG-rich lipoproteins by the liver. Studies have demonstrated an increase in fatty acid intestinal secretion accompanied by elevated chylomicron secretion in obesity. This increased chylomicron secretion leads to the increased delivery of fatty acids to the liver.

The increase in liver fatty acids via these three pathways results in increased TG liver synthesis. VLDL formation and secretion str related to Apo B-100 degradation protection. In addition, the ability of insulin to suppress Apo B secretion is impaired in obese patients and is marked by insulin resistance. Lastly, increased caloric intake can contribute to TG circulation, either by food fats that cause a rise in the chylomicron levels and/or by fatty acids being provided to the liver, or by a carbohydrate-rich diet that increases hepatic de novo lipogenesis [25].

In addition to TG-rich lipoprotein overproduction by the liver and intestine, there are subsequent metabolic abnormalities of these TG-rich lipoproteins, which contribute to increased TG levels. Obese patients have increased Apo C-III levels. Apo C-III expression is inhibited by insulin and, therefore, obese patients’ insulin resistance could explain the Apo C-III increase [27]. Apo C-III is a lipoprotein lipase inhibitor and, therefore, could reduce the clearance of TG-rich lipoproteins. Furthermore, Apo C-III inhibits TG-rich lipoprotein cellular uptake [25].

Increased TG-rich lipoprotein levels have, in turn, effects on other lipoproteins. Specifically, cholesterol ester transfer protein (CETP) mediates the equimolar exchange of TG-rich VLDL triglycerides and chylomicrons with LDL and HDL cholesterol. Increased TG-rich lipoproteins lead to increased CETP-mediated exchange, causing an increased TG content and decreased cholesterol content of both LDL and HDL [25]. Moreover, obesity increases CETP activity and levels [28]. This CETP-mediated exchange is the basis of the reciprocal relationship observed between low HDL cholesterol concentrations when TG levels are high and increased HDL cholesterol when TG levels decrease [25].

TG contained by LDL and HDL is later hydrolyzed by hepatic lipase and lipoprotein lipase, resulting in small, dense LDL and HDL particles. Hepatic lipase activity is increased in patients with excessive visceral fat, which facilitates LDL and HDL triglyceride removal, resulting in small lipoprotein particles. The affinity of Apo A-I for small HDL particles is reduced, leading to Apo A-I dissociation, discomposure, and kidney elimination. These processes lead to decreased Apo A-I and HDL cholesterol levels in obese patients [25]. Lipolysis, in obese patients, is affected by low lipoprotein lipase messenger ribonucleic acid (mRNA) expression in the adipose tissue and reduced lipoprotein lipase activity in the skeletal muscle [24].

Chronic inflammation, a well-known aspect of obesity, can affect the adipocytes’ cholesterol efflux capacity, thus reducing plasma HDL cholesterol levels [26]. Cytokines produced by macrophages and adipokines produced by adipose cells also modify the lipid metabolism. Adipokines, such as adiponectin and resistin, regulate the lipid metabolism. Circulating adiponectin levels are low in obese patients and are associated with increased serum TG and decreased HDL cholesterol levels. Resistin is increased in obese patients, and its levels directly correlate with plasma TG levels. Moreover, it has been demonstrated that resistin stimulates hepatic VLDL production and secretion due to increased Apo B, TG, and cholesterol synthesis. Finally, resistin is associated with decreased HDL cholesterol and Apo A-I levels [25].

Proinflammatory cytokines, tumor necrosis factor (TNF), and interleukin (IL)-1 stimulate lipolysis in the adipocytes and increase FFA circulating levels, which offer hepatic TG synthesis substrate. Inside the liver, pro-inflammatory cytokines stimulate de novo fatty acid and triglyceride synthesis. These alterations increase VLDL production and secretion. Pro-inflammatory cytokines also decrease lipoprotein lipase excretion and increase angiopoietin expression, as well as protein 4, a lipoprotein lipase inhibitor. Together, these alterations decrease lipoprotein lipase activity, thus delaying TG-rich lipoprotein elimination. Thus, increased pro-inflammatory cytokine levels stimulate TG-rich lipoprotein production and delay TG-rich lipoprotein elimination, leading to the increased serum TG noticed in obese patients. Inflammation also diminishes other important HDL functions, such as its ability to prevent LDL oxidation [25].

The term “oxidative stress” can also be related to the pathogenesis of obesity, which determines the lipoprotein modifications, making them dysfunctional and more atherogenic [29]. Different types of modified lipoproteins promote the development of the inflammatory process within the arterial wall, macrophage activation, and the synthesis and secretion of proinflammatory cytokines, chemokines, and enzymes. Of these, glycated lipoproteins are characterized by greater atherogenicity [30].

## 4. Current Therapy Approach

As expected, obesity and dyslipidemia are closely correlated, being the main features of metabolic syndrome and the basis of its pathogenic mechanisms through adipose tissue dysfunction. The increasing prevalence of metabolic syndrome appears to be closely linked to the obesity epidemic, and this, in turn, is correlated with the increasing prevalence of dyslipidemia, which means that a thorough understanding of these two entities could aid in the formulation of future therapeutic approaches. Dyslipidemia is a major risk factor for cardiovascular disease, which is the leading cause of death worldwide. This unwelcome situation exemplifies the urgent need for an effective solution to the problem [19].

The attempt to find effective solutions to obesity has been at the heart of research for decades, as its incidence continues to grow at an accelerated rate, with major effects on an already overworked health system and the worrying consequence of an increasing global mortality rate [31]. The first step in proper management of obesity is to recognize obesity as a chronic disease which is associated with various comorbidities, and its successful management often requires adjuvant pharmacological interventions aiming to strengthen the necessary behavioral strategies [32].

Improving obesity-related medical conditions can be achieved through a moderate, sustained decrease in body weight. While significant research has focused on developing new and improved nutritional and dietary approaches to energy intake regulation, adjusting the public opinion on diet and food choices and promoting a healthy lifestyle have proven to be difficult tasks [33].

Current clinical treatments for obesity focus on pharmacotherapy and surgery [34]. The successful use of obesity-targeting pharmacotherapy requires the careful, patient-centered discussion of topics such as food and social behavior, medical history, financial preferences, and expectations, as well as data on the known side effects of medications that may alter a patient’s lifestyle and quality of life [32]. Currently approved drugs that are used for dyslipidemia and obesity include orlistat, lorcaserine, liraglutide, phentermine/topiramate, fibrates, statins, niacin, resins, ezetimibe, sibutramine, bupropion, and naltrexone. Each of these medications is accompanied by a number of side effects, such as discomfort or abdominal pain, nausea, insomnia, constipation, headaches, and vomiting [34].

Bariatric–metabolic surgery is the most effective treatment option for clinically severe obesity, providing significant and lasting weight loss results. The subsequent weight loss offers ample benefits, including reduced mortality (especially due to cardiovascular disease, diabetes, and cancer) and a better quality of life [35]. However, these treatments are often accompanied by side effects, such as intestinal bleeding and, in rare cases, suicide.

Consequently, this explains the need to identify new therapeutic approaches for the treatment or prevention of obesity and metabolic syndrome. Attempts are being made to pay more attention to the design of safe, inexpensive, and highly effective materials that control the bioavailability of fats and carbohydrates in order to successfully manage the nutritional value of foods and to combat the comorbidities associated with obesity. Thus, this led to the evaluation of various biomaterials, from inorganic colloids to organic polymers, the focus of study being their ability to interfere with digestive mechanisms. One of the objectives is the orientation of research towards the development of new therapies and nutraceuticals that systematically control the nutritional value of foods through colloidal engineering approaches, the use of such materials in the clinical environment being essential for combating obesity [33].

Genetic and epigenetic therapies are two possibilities that are focuses of current explorations, but their supply through the traditional methods of viral vectors is accompanied by low effectiveness, requiring a technology that can encapsulate the material to protect it from degradation and absorption at the level of the circulating reticuloendothelial system. These include physical methods that inject genetic/epigenetic materials directly into the host cell, as well as chemical methods that surround the materials with protective molecules, such as nanotechnology methods [17].

## 5. Nanotechnology and Dyslipidemia in Obesity: An Old Disease with Innovative Treatment Strategies

The subject of nanotechnology, labeled as “tiny science”, has grown in recent years, being at the center of the medical stage. Dating back to 4500 years ago, to the use of natural asbestos nanofibers for ceramic matrices, nanotechnology has made an impressive journey through, history with notable prospects for the production of new materials in the field, where conventional approaches have reached their limit.

Nanotechnology refers manly to objects between 0.1 and 100 nm that are made of metal oxides, carbon, organic matter, and so on, with multiple positive attributes such as: (i) cost-effectiveness, (ii) environmentally friendly status, (iii) a high surface charge, (iv) a vast surface area, (v) absorption capacity, (vi) chemical reactivity, (vii) sensitivity, (viii) stability, (ix) mechanical strength, and (x) biological activity. The field of nanomaterials focuses mostly on nanoparticles, as the essential building components of nanotechnology, but also on other nanostructures, such as as nanocomposites, nanotubes, nanospheres, nanocapsules, nanorods, nanowires, nanofibers, nanorings, nanofilms, nanocrystals, and nanostars. More details about low-dimensional nanomaterials and their distinctive features can be found in the numerous review papers [36,37] or book chapters [38] available on the PubMed database.

During the last decade, the development of nanotechnology applications for obesity therapies has attracted the attention of many research groups. The benefits granted by nanotechnologies reside in their potential to overcome the side effects and the inefficiency of classic therapies, promoting better compatibility or enhancing biological processes [39].

Sandhu et al., in their recent review paper [40], detailed the advantages and disadvantages of nanotechnology used in medicine, a summary of which is briefly presented below, without repeating from the above-written attributes. Among the advantages, we remark on the following: tunable surface properties and size, controlled and targeted release, and the versatility of the administration route. The disadvantages are related to the aggregation potential, which leads to the deterioration of the nano-formulation, and the drug loading capacity’s dependence on size.

### 5.1. Nano-Formulations and/or Drug-Loaded Nanocarrier

As mentioned above, the pharmacological therapy for obesity, which includes drugs such as orlistat, rimonabant, sibutramine, dexfenfluramine, fenfluramine, or phenyl propylamine, has a low efficacy and produces systemic drug toxicity and multiple side effects related to mental disorders and non-fatal myocardial infarction, as well as stroke, depression, and anxiety, therefore limiting their use [41]. To overcome these limitations, innovative drug delivery carriers, such as nanoparticles based on gold, phosphatidylcholine, and cholesterol; PLGA-b-PEG, PLGA, dextran, and dextran-PEG; liposomes based on peptides; nano-emulsions based on Capryol PGMC and Cremophor RH40; and lipase-sensitive nanocarriers were formulated in order to enhance the therapeutic efficacy of the synthetic drugs, providing targeted action (as presented in Table 2).

In 2018, Zhang et al. [54] evaluated recent advances in nanomedicine as an emerging strategy for obesity treatment from two points of view, one related to the suppression of digestibility and another to the enhancement of energy expenditure. The authors pointed out that even if administered nanocarriers offer an efficient approach to body weight control, different aspects still remain challenging, namely our understanding of the routes involved in the regulation of energy homeostasis, the lowering of the side effects, the evaluation of the biocompatibility in long-term administration, and, of course, successful clinical trials and further marketization.

In 2019, Sibuyi et al. [55] described nanotechnology-based therapies as an alternative strategy that can be used to treat obesity and overcome the downsides associated with usual therapies using three different strategies, namely: (i) the inhibition of angiogenesis in the white adipose tissues (WATs); (ii) the transformation of WATs into brown adipose tissues (BATs); and (iii) the photothermal lipolysis of WATs. The summarized nanocarriers (liposomes and polymeric and gold nanoparticles) showed a high tolerability, reduced side effects, and enhanced efficacy in a reproducible manner, thus providing proof of concept for the hypothesis that targeted nanotherapy can act as a feasible tactic for combatting obesity and prevent its comorbidities. A chapter in this review paper that is worth mentioning is related to the nanodrugs used in clinical practice. Until 2019, there were no nano-based drugs that were either clinically approved or in clinical trials for the treatment of obesity. However, the authors assumed that it was only a matter of time before the identified nanodrugs in the literature would be tested in clinical trials and made available to the market. However, based on a simple search of the ClinicalTrials.gov platform using nanomaterials and obesity as search terms, there have still been no related trials after 3 years.

However, it is clear that the drug-based treatment of obesity or dyslipidemia is considered as long-term therapy, since most patients regain weight upon stopping medication. Therefore, we must consider whether the nano-systems alone also have positive effects on weight loss and lipid profiles when used as the synthetic drug but without its side effects. Table 3 presents several nanostructures, such as nanoparticles based on superparamagnetic iron oxide grafted with carboxyethylsilanetriol, cerium oxide, chitosan, and water-soluble chitosan and silica, nanorods based on gold, and nanospheres based on gold and hyaluronate, and their therapeutic roles in the management of obesity. Even if more work is required regarding longer periods of treatment and the testing of normal-weight subjects in order to shed further light on the modes of action of bare nanomaterials in obesity-related disorders, these results should also be taken into consideration in the development of alternative drug delivery systems.

The current review also proposes the impacts of nanotherapy on the driving forces of dyslipidemia, namely ectopic lipid deposition, adverse lipid metabolism, and dysfunctional adipose tissue [63]. It is well known that obesity and dyslipidemia are considered as two disorders that go together, but the question is: are there any related studies using nanomaterials for dyslipidemia therapy?

The search of the PubMed.gov database returned nearly 60 papers using nanomaterials and dyslipidemia. By analyzing these papers, we can see that only a few used synthetic drugs specifically for dyslipidemia combined with nanostructures based on selenium, coenzyme Q10, vitamin E, glyceryl monostearate, Poloxamer 407, didodecyldimethylammonium bromide, and β-cyclodextrin cross-linked with diphenyl carbonate, palmityl alcohol, and Tween 40/Span 40/Myrj 52, as presented in Table 4, while more used natural compounds mixtures, as described in next section.

However, to date, no commercial product based on nanotechnology combined with disease-specific drugs has been developed for dyslipidemia treatment, and further investigations are necessary.

As observed from the gathered data in Table 2, Table 3 and Table 4, significant advances have been made towards the targeting of adipose tissue and related disorders. However, there is still potential to improve the efficacy of the nano-based formulations. There are also many questions that have not been fully addressed [71], including: (i) the formulation and optimization of the loaded nanocarrier; (ii) bioavailability studies; (iii) the elucidation of the relationship between dysfunctional cells and nanomaterials; (iv) preclinical evaluations of long-term toxicity, targeted delivery, metabolism, and pharmacokinetic and pharmacodynamic assays; and (v) the real-time monitoring of the diseases during treatment.

### 5.2. Herbal Nanotherapy

Herbs are preferred by many people over conventional medications as a means of relieving their disease symptoms. As a result, the recent pharmaceutical industry focused on discovering novel therapeutic agents from medicinal plants. About 25% of the natural new molecular entities approved by the Food and Drug Administration (FDA) had plant origins in 2020 [72].

The tremendous interest in the use of traditional medicines worldwide has various reasons, namely the facts that they are affordable, have an increased availability, are natural and safe, with low to no adverse effects, are compatible with the patient’s ideology, offer personalized health care, and enable more public access to health information. Even if herbal medicines are used in health promotion and therapy for chronic diseases, their usage intensifies when conventional medicine is ineffective, as in the treatment of advanced cancers or new infectious diseases [73].

In the current scenario, the use of plants and active phytochemical constituents acts as a valuable foundation for the development of novel anti-obesity products. Yet, there are still numerous facts that should be noted when using herbal medicines, such as the fact that their efficacy is still an important point that must be elucidated for the purpose of interpretation, and the facts that their safety still remains to be evaluated and their anti-obesity mechanisms are still not entirely clear. However, herbal medicines are considered an effective strategy for the management of obesity and associated disorders due to their identified mechanisms of action [74], which are as follows: (i) the regulation of the plasma lipid profile; (ii) inhibition of pancreatic lipase activity; (iii) regulation of gene expression and leptin levels and activity; (iv) reduction in the accumulation of white adipose tissues; (v) inhibition of α-amylase, ghrelin, and α-glucosidase; (vi) increase in the expression of peroxisome proliferator-activated receptors (PPAR) α, β, and γ; (vii) regulation of mRNA-mediated lipid metabolism; (viii) increase in the levels of adiponectin and hepatic Co-A oxidase activity; (ix) the balance of energy intake and expenditure; (x) suppression of adipocyte maturation and differentiation; (xi) decrease in body weight gain; and (xii) the reduction of stress.

There are numerous medicinal plants that have been examined for the treatment and management of obesity, as detailed in many review and original articles. There are nearly 300 papers available on PubMed.gov database using obesity and herbs as keywords, from 1965 and up to the current date (June 2022), with an increasing trend in the last 7 years, emphasizing the necessity to develop alternative strategies for the treatment of obesity.

Obesity, dyslipidemia, and diabetes mellitus are among the diseases most frequently “treated” with medicinal plants [75], such as the Chinese herbal medicine formula (RCM-104) [76], evaluated in a double-blind, randomized, placebo-controlled trial on obese subjects, or bofu-tsusho-san (an oriental herbal medicine) [77] in a trial conducted on Japanese women with impaired glucose tolerance in the first randomized, double-blind, placebo-controlled study.

Cambodia hoodia, Citrus aurantium, caffeine, capsaicin, chitosan, ephedrine, green tea, guar gum, fenugreek, white beans, yohimbine, and fitostreols were studied in clinical trials up to 2015, and their anti-obesity properties were mainly determined to be a strong antioxidant activity [78]. As expected, these medicinal plants have been recommended for weight loss, but more studies are needed to determine their effectiveness, safety, and pharmaceutically active ingredients that contribute to this weight loss property. Overall, the presented data supports the usage of herbal medicines in most societies.

A cross-sectional study conducted in 2017 on overweight and obese people in Taif, Saudi Arabia, using a pretested questionnaire demonstrated that 98.1% of patients used herbal medicines to lose weight, with either green tea (88.4%) or ginger (29.5%) being the most commonly used herbs, which were prescribed mainly by a friend (35.8%) or herbalists (31%), with 35.5% of patients revealing side effects. This was first study that investigated the use of herbal medicines in obese or overweight people in the Taif area and determined that 72.0% of the participants would continue to use herbal plants in the future. However, further studies on the benefits and risks are needed, along with an efficient community-based awareness program to broadcast these alternative therapies for weight loss [79].

The article of Liu et al. [80] from the same year, 2017, discusses, among the possible effects and mechanisms of herbal medicine, the treatments for obesity reported in the period from 2007–2017 and the situation concerning the clinical trials, using different keywords: obesity, herbal medicine, plant, and Chinese medicine. There were 18 published randomized controlled trials and 16 registered clinical trials of herbal medicines for the treatment of obesity in humans from 2007 to 2017.

The review of Karri et al. [74], assessing research conducted from 2000–2018, collected the facts related to natural anti-obesity agents, detailing their biological sources, chemical constituents, and probable mechanisms of action. Among the identified natural isolated constituents that showed satisfactory anti-obesity properties, we mention only a few, namely curcumin, hydroxycitric acid, apigenin-7-O-D-glucoside, carnitine, polyphenols, ginseng crude saponins, crocin, capsaicin, mangiferin, flavonoids, berberine, carnosic acid and carnosol, anthocyanins, gingerol, paradol, shagol, rutin, resveratrol, phlorotannins, agavins, camphor, benzaldehyde, caffeine, polysaccharides, tannins, celastrol—a triterpene, fructan, gallic acid, catechins, xanthines, purine alkaloids, quercetin, kaempherol, chlorogenic acid, eugenol, acetyl eugenol, caryophyllene, humulene, fatty acid synthase, scopoletin, and so on. More details can be found in the above-mentioned review paper.

However, after several years of constant research on this topic, the main drawback still applies, namely, the fact that the possible pharmacological and therapeutic mechanism is not completely understood, and there is still room for additional contributions. Therefore, our main query refers to the development of alternative, naturally based anti-obesity treatments that are widely increasing in terms of their species, extract types, and so on, but that are still not validated as being effective or safe.

We return not to the subject of this review chapter, namely, nanotherapy based on natural products, which is considered to be a new alternative for conventional obesity treatments. The latest review from 2021 by Shende and Narvenker [81] also details the nanocarriers used for the delivery of herbal drugs, namely liposomes, solid lipid nanoparticles, phytosomes, polymeric and magnetic nanoparticles, nanosized micelles, and gold nanoparticles.

Yet, why should we use nanotechnology when there are numerous clinical approaches that recommend the extraction and use of natural products as anti-obesity agents?

As mentioned above, nanomaterials overcome the limitations and side effects of the classical treatments [82] but also resolve several issues of the phytoconstituents, namely: (i) the increase in the bioavailability and stability; (ii) protection of the active natural extract from chemical degradation in the body; (iii) the reduction in the correlated toxicity, if any; (iv) the enhancement of the pharmacological activity; (v) provision of a sustained and controlled release; (vi) an increased targeting efficiency; (vii) an enhanced specificity; (viii) the ability to overcome the hindrances of herbal medication; and (ix) a diminished administration frequency and dose [81].

Table 5 presents several nanocarriers based on gold, silver, ligand-coated R, poly-vinyl alcohol gelatin, alginate, chitosan, lipids, monoolein, and PLGA used for formulating herbal nanotherapeutics that demonstrate hypolipidemic, hypoglycemic, and antioxidant properties as suitable complements to the current anti-obesity drugs.

A different tableau is observed in dyslipidemia, one of the major metabolic disorders, with the following abnormalities: (i) higher levels of Apo B, triglycerides, LDL-cholestrol, and free fatty acids; (ii) very-low-density lipoproteins; (iii) low-density lipoproteins; and (iv) decreased levels of high-density lipoproteins [100], which could be resolved using natural products or herbal nanotherapy.

The review paper of Khutami et al. [101] discusses the main phytoconstituents with antioxidant activities that are used to tackle dyslipidemia via several mechanisms, such as resveratrol, curcumin, quercetin, anthocyanin, antioxidant polysaccharide okra, green tea, strawberry ellagitannins, α-terpineol, and other antioxidants. As observed, this list is similar to that of the drugs used to tackle obesity, as expected, since there is a clear relationship between these metabolic disorders and the effects of antioxidants.

According to a recent study, Bergamot-derived extract, containing high amounts of flavonoids (neoeriocitrin, neohesperidin, and naringin), reduces the serum levels of lipids and improves the lipoprotein profile, as shown in 80 subjects with moderate hypercholesterolemia. This novel study administered Bergamot-derived extract at a fixed daily dose (150 mg of flavonoids, with 16% neoeriocitrin, 47% neohesperidin, and 37% naringin) for 6 months. The results revealed a reduction in the carotid intima media thickness over the investigated timeframe [102]. In a randomized double-blind placebo-controlled crossover trial, 30 obese dyslipidemic subjects were given curcuminoids (1 g/day) or a placebo for 4 weeks, followed by a 2-week break and then treatment with the alternate drug for another 4 weeks. Even if the results showed that supplementation with curcuminoids did not cause any significant alteration to the serum small dense low-density lipoprotein [103], the authors encouraged the exploration of the impacts of curcuminoids on the serum lipidome, triglycerides and fatty acid compositions of lipoprotein species. In another meta-analysis of randomized controlled trials, different flaxseed products (whole flaxseed, oil, and lignans) are evaluated for their effects on the lipid profiles and inflammatory and anthropometric parameters of patients with dyslipidemia-related diseases. Whole flaxseed and lignans reduced the blood lipid levels, flaxseed oil had an important anti-inflammatory activity, and whole flaxseed lowered the lipid profile and weight [104]. Therefore, the study suggested that these natural product extracts are promising candidates for the treatment of dyslipidemia. In the following Table 6, we analyze the natural products encapsulated in nano-systems, such as nano-emulsion with Tween, nanoparticles made of gold, silver, selenium, and chitosan, and liposomal nano-formulations with antioxidant and anti-hyperlipidemic effects.

Overall, as observed in Table 5 and Table 6, different nano-systems have been designed using compounds of plant origins in obesity and/or dyslipidemia therapies, most of them having a similar composition of the nanostructure or plant extract. The obtained results highlighted their impacts on the disease pathways, lipid profile, oxidative stress, biochemical markers, and so on, with a great potential to be used as alternative therapies in clinical practice. However, even if herbal nanotherapy has a positive outlook as a metabolic disease treatment, there is still an open path to the evaluation and validation of the therapeutic efficacy and safety of the phytoconstituents, a route that is travelled, unfortunately, in small steps. Moreover, what if the nano-formulations of the drugs already in use were to be supplemented with phytoconstituents in order to increase the drugs’ bioavailability, providing better in-vivo efficacy and reduced toxicity in treating hyperlipidemia? This approach appears to be beneficial, according to a study [65] from 2018, where CoQ10 and Vitamin E supplementations eliminated statin myopathy and liver injury. However, of course, further research using preclinical studies and controlled trials are needed in order to overcome the limitations of the drugs used in parallel with plant extracts as new drug delivery systems.

## 6. Conclusions

Obesity and dyslipidemia denote serious health concerns as high risk factors of severe chronic diseases, such as type-2 diabetes and cardiovascular diseases. In addition to lifestyle modification and surgery, pharmacotherapy remains the main pillar of the management of metabolic-syndrome-related diseases. As expected, the necessity of an effective strategy for treating the pathophysiology has led to an unmatched expansion in the development of various kinds of nanostructures for obesity- and dyslipidemia-related therapies.

The current status, challenges, and future perspectives on targeted nano-systems were discussed in this short review. However, despite the extensive research on the therapeutic effects of either bare nano-formulations or those that have been integrated with drugs that are already in use or with plant-derived bioactive compounds, their delivery, safety, efficacy, and bioavailability are still problematic. Therefore, the development of anti-obesity/dyslipidemic nanoparticulate agents that are disease-specific, safe, simple to formulate, and yet still dominant, will continue to be the focus in the near future, offering these drugs greater potential to be translated into the clinical setting.

## Figures and Tables

**Table 1:** Dyslipidemia clinical profile.

Diagnostic Criteria of Dyslipidemia	References
Increased TG and FFA	[23]
HDL dysfunction	
Decreased HDL cholesterol	[24]
Normal or slightly elevated LDL cholesterol or LDL formation	
Increased VLDL cholesterol or overproduction by liver	[25]
Apo B concentrations elevated, partially due to hepatic overproduction	[24]
Low Apo A-I levels	[25]
Low HDL-levels	[26]
Decreased circulating TG lipolysis	[24]
Impaired peripheral FFA uptake	[25]
Insulin resistance and macrophage infiltration of the adipose tissue, inducing a pro-inflammatory status	

TG: triglyceride; FFA: free fatty acids; HDL: high-density lipoprotein; VLDL: very-low-density lipoprotein; LDL: low-density lipoprotein; Apo: apolipoprotein.

**Table 2:** Drug-based nanostructures and their therapeutic roles in the management of obesity.

Nanostructure Type	Drug	Observed Effects	References
Gold nanoparticles (<50 nm)	Adipose homing peptide	Selective targeted delivery on white adipose tissue vasculature in vivo.	[42]
Prohibitin-targeted nanoparticles with PEG chains (109.2 ± 7.8 nm)	Proapoptotic peptide	Reduces weight gain via the control of the adipose function by 14%.	[43]
Prohibitin-targeted nanoparticles (around 100 nm)	Cytochrome C	Prevents diet-induced obesity in C57BL/6 mice in a dose-dependent manner; effectively targeted the adipose tissues and the Cytochrome C released at the adipose site from the nanoparticles; caused apoptosis of the adipose cells.	[44]
Egg-yolk-phosphatidylcholine- and cholesterol-based nanoparticle-conjugated PEG-lipids (around 130 nm)	Prohibitin-targeting peptide	Reduces undesired entrapment in liver and, hence, improves the efficient targeting of adipose vessels.	[45]
Peptide-ligand-mediated nanocarrier (lipopeptide-modified liposomes of 105.6 ± 13.9 nm)	Linear peptide, followed by an adipose tissue-specific circular peptide (KGGRAKD)	Successful delivery of the aqueous phase to the cytoplasm of primary cultured endothelial cells derived from inguinal adipose tissue.	[46]
PLGA-b-PEG nanoparticles (about 100 nm)	Endothelial-targeted peptides (iRGD and P3)	Weight gain inhibition was confirmed in the diet-induced obese mouse model.	[47]
Nano-emulsion based on Capryol PGMC and Cremophor RH40 (139.4 ± 12 nm)	Orlistat	Overcomes high lipophilicity, improves dissolution and pancreatic lipase inhibition in vivo	[48]
Lipase-sensitive nanocarrier (self-assembled amphiphilic copolymer BTTPFN-g-PCL; 158 nm)	Orlistat	Lowers weight of the liver or fat pads, smaller adipocyte size, and lower total cholesterol level.	[49]
PLGA-b- PEG-triphenylphosphonium polymer nanoparticles (∼80 to ∼410 nm)	Mitochondrial decoupler 2,4-dinitrophenol	Reduces lipid accumulation in the adipocytes, but may also lead to the excessive generation of reactive oxygen species and its possible impairment in non-adipose tissues.	[50]
PLGA nanoparticles (177 ± 6 nm)	Dibenzazepine	Browning of adipocytes, consequently improving glucose homeostasis and attenuating body weight gain in the treated diet-induced obese mice.	[51]
Dextran and dextran-PEG nano-carriers (4–30 nm)	Dexamethasone	Restored the gene expression of key pro-inflammatory cytokines (TNFα, IL-6, MCP-1) and ameliorated many critical effects of obesity-induced inflammation.	[52]
Polymeric nanoparticles (200 nm)	Rosiglitazone	Alleviated inflammatory reactions in the white adipose tissue and liver.	[53]

PEG: polyethylene glycol; PLGA: poly (lactic-co-glycolic acid); PGMC: propylene glycol monocaprylate; PCL: polycaprolactone.

**Table 3:** Nanostructures and their therapeutic roles in the management of obesity.

Nanostructure Type	Observed Effects	References
Superparamagnetic iron oxide nanoparticles grafted with carboxyethylsilanetriol (very narrow size distribution of <20 nm)	Downregulated the expression of 22 and 29 risk genes and the mRNA expression of lipid and glucose metabolism genes upon exposure to human primary adipocytes.	[56]
Cerium oxide nanoparticles (5–80 nm)	Reduced the weight gain and lowered the plasma levels of insulin, leptin, glucose, and triglycerides.	[57]
Chitosan and water-soluble chitosan microparticles and nanoparticles	A significantly lower degree of weight gain in a high-fat-diet rat model, reduced the final amounts of epididymal and perirenal white adipose tissues, liver weight, total serum cholesterol, and low-density lipoprotein cholesterol.	[58]
Carboxyethylsilanetriol grafted superparamagnetic iron oxide nanoparticles (<10 nm)	Crucial dual role in the expression of 22 and 29 risk genes (based on gene-wide association studies) for obesity and T2DM in human adipocytes.	[56]
Silica mesoporous particles (2D hexagonal pores)	Decrease in weight and body fat composition without observable toxicological signs or systemic absorption of silica.	[59]
Gold nanorods energized by an external near-infrared exposure at 800 nm (NanoLipo)	Disruptions in the adipose tissue and removal of 33% subcutaneous tissue and ~60% free fatty acids, leading to a great decrease in the adipose layer thickness at 1 month post-surgery.	[60]
Hyaluronate gold nanosphere conjugated with photothermal lipolysis	Enables the highly effective photothermal ablation of adipose tissues in C57BL/6 obese mice, successful transdermal delivery, and photothermal lipolysis.	[61]
Gold nanoparticles (21 nm)	An 8% or 5% reduction in body weight, improved hyperlipidemia, and normal glucose tolerance.	[62]

~: approximately.

**Table 4:** Drug-based nanostructures and their therapeutic roles in the management of dyslipidemia.

Nanostructure Type	Drug	Observed Effects	References
Nano-selenium	Atorvastatin	Significantly reduced serum TC, TG, and LDL-C contents; declined tissue lesions, such as the aortic arch and liver; enhanced the activities of GPx-1 and SOD in the serum; decreased the MDA content; and increased the SOD activity in rat aorta.	[64]
Nano-particulate formulation (nano coenzyme Q10 and nano-vitamin E)		Reduction in the number of liver and muscle enzymes and histopathological alterations, together with a marked decline in oxidative stress.	[65]
Solid-lipid nanoparticles (glyceryl monostearate, Poloxamer 407; 88.91 + 1.23 nm)		All nano-systems showed increased bioavailability. The nano-sponges were found to be an excellent carrier of the drug, providing a sustained drug release over a prolonged period of time, lowering the LDL, TC, and TG, and increasing the HDL over a period of 7 days.	[66]
Nanocrystals (based on didodecyldimethylammonium bromide; 139.6 + 2.21 nm)			
Nano-sponges (β-cyclodextrin cross-linked with diphenyl carbonate; 298.2 + 1.02 nm)			
Nanostructured lipid carriers (187.6 ± 3.04 nm)	Simvastatin	Enhanced bioavailability and improved biological efficiency of the drug; improved plasma and erythrocyte membrane lipids; maintenance of the erythrocyte oxidant/antioxidant balance; and decreased hemolysis in hyperlipidemic conditions.	[67]
Solid lipid nanoparticles (palmityl alcohol, Tween 40/Span 40/Myrj 52; ∼130 nm)		Sustained release, significantly reduced the elevated serum lipids, and decreased total cholesterol in hyperlipidemic rats.	[68]
Solidified self-nano-emulsifying drug-delivery system (∼100 nm)	Rosuvastatin	Improved drug release (∼95%), reduction in cholesterol, triglyceride, and atherogenic indices, and increased high-density lipoprotein levels.	[69]
Nanoliposomes (negatively charged surface)	-	Decreased triglyceride, total cholesterol, and low-density lipoprotein cholesterol levels, and increased high-density lipoprotein cholesterol in high-cholesterol-diet rabbits.	[70]

TC: total cholesterol; TG: triglycerides; SOD: superoxide dismutase; MDA: malondialdehyde.

**Table 5:** Natural anti-obesity nanostructures and their therapeutic roles in the management of obesity.

Nanostructure Type	Natural Product	Observed Effects	References
Gold nanoparticles (20–50 nm with a spherical morphology and crystalline nature)	Salacia chinensis	Decreased the body weight, resistin, liver marker enzymes, leptin, adipose index, and inflammatory markers; increased the levels of high-density lipoprotein, AMP-activated protein kinase, and adiponectin.	[83]
Gold nanoparticles (hollow spheres of 50–90 nm)	Smilax glabra rhizome	Both anti-obesity and anti-diabetic effects: mediate glucose and insulin discharge; normalize the liver markers, lipid profiles, body weight, body mass index, and hormone profile.	[84]
Gold nanoparticles (spherical, poly-dispersed, of 20 nm)	Poria cocos	Reduce the weight gain and body mass index, regulate glucose and lipid metabolism, inhibit adipose tissue inflammation, scavenge oxidative stress, and normalize the satiety hormones.	[85]
Silver nanoparticles (spherical, of 15 nm)	Argyreia nervosa	Inhibitory activity against carbohydrate digestive enzymes α-amylase and α-glucosidase; strong antibacterial activity against foodborne bacteria, *Escherichia coli* and *Staphylococcus aureus*.	[86]
Ligand-coated R-encapsulated nanoparticles (L-Rnano) (spherical shape with a size of 90–110 nm)	Resveratrol	Induced adipose stromal cell differentiation into beige adipocytes, reduced the 40% fat mass and inflammation, and enhanced glucose hemostasis.	[87]
Poly-vinyl alcohol gelatin nanofibers (200 to 250 nm)	Curcumin	Reduce the number of adipose tissues by up to 4–7% in model rats.	[88]
Phosphatidylcholine phytosome nanoparticles (51.66–667.24 nm) and phytosome thermogel	Soybean seed extract	Reduction in body weight, adipose tissue weight, and lipid profile.	[89]
Single-layer nano-emulsion and alginate double-layer nano-emulsion	Oleoresin capsicum	Inhibits intracellular lipid accumulation and triglyceride content and enhances the release of free fatty acids and glycerol into the medium	[90]
Lipid-derived nano-vesicles (<50 nm and >150 nm)	Citrus sinensis	Increased villi size, reduced triglyceride content, and modulated mRNA levels of TNF-α and IL-1β genes, barrier permeability, fat absorption, and chylomicron release.	[91]
Gold nanoparticles (size 10–20 nm)	Dendropanax morbifera	Reduced triglyceride content, down-regulated PPAR-γ, CEBPα, Jak2, STAT3, and ap2 expression in 3T3-L1 cells and FAS and acetyl ACC levels in HepG2 cells.	[92]
Lipid nanocarriers (200 nm)	Capsaicin	Decrease in body weight by up to 15% as compared to the control and improved lipid and glucose profiles.	[93]
Lipid nanocarriers and liposomes (140 nm and 110)	Resveratrol	Enhanced uncoupling protein 1 and beige marker CD137 expression.	[94]
Self-assembly and directed assembly of lipid nanocarriers	Silibinin	Prevents liver fibrosis in obese rats, enhanced bioavailability 2.9 fold, and improved therapeutic action.	[95]
Solid lipid nanoparticles	Hydroxycitric acid	Improved bioavailability, enhanced the pharmacological action, provided a targeted delivery to the adipose tissues, and reduced the associated side effects.	[96]
Chitosan nanoparticles (210 nm)	Chlorogenic acid	Sustained release property, retained antioxidant activity, and enhanced bioavailability.	[97]
Cubic phase monoolein nanoparticles (205–295 nm)	Grape, apple, mugwort, barberry root, and green tea extracts	Decreased the blood contents of aspartate aminotransferase, total cholesterol, triglyceride, urea nitrogen, and low-density lipoprotein; promoted the efficacy of the herbal extracts in suppressing body weight gain and liver weight gain in rats.	[98]
PLGA nanoparticles (Nano-Orz, 214.8 ± 4.3 nm)	γ-Oryzanol	Ameliorated fuel metabolism, with an excellent impact on the dysfunction of the hypothalamus and pancreatic islets; decreased ER stress and inflammation in the liver and adipose tissue.	[99]

FAS: fatty acid synthase; ACC: CoA-carboxylase.

**Table 6:** Natural nanostructures and their therapeutic roles in the management of dyslipidemia.

Nanostructure Type	Natural Product	Observed Effects	Reference
Nano-emulsion with Tween (24.9 ± 1.11 nm)	Garlic oil	Significant effect in lowering the lipid profile and the lipid deposits in hepatic tissues.	[105]
Gold nanoparticles (smooth spherical morphology with 7–27 nm)	Ziziphus jujube	Significant decrease in the levels of liver, insulin, triglycerides, cholesterol, and total antioxidant capacity.	[106]
Oil-in-water nano-emulsion (133.4 ± 0.2 nm)	*Hibiscus cannabinus L*.	Declined accumulation of fat droplets in the liver, lowered cholesterol, decreased number of endogenous antioxidants in the liver, and controlled weight in high-cholesterol-diet-induced rats, with the accelerated renewal of liver cells after injury.	[107]
Selenium nanoparticles (spherical crystals of 18–50 nm)	Black currant	Increased hypolipidemia antioxidant activity in galactose-treated rats	[108]
Silver nanoparticles (200 nm)	Nigella Sativa	Decreased levels of triglycerides, cholesterol absorption, low-density lipoproteins, oxidative stress, and increased high-density lipoproteins.	[109]
Chitosan nanocarrier	Fennel, rosemary volatile oils	Reduced dyslipidemia and CVDs risk, improved liver dysfunction, lowered MDA and TNF-α and blood sugar values.	[110]
Self-nano-emulsifying delivery system (48% surfactant Kolliphor and 12% co-surfactant PEG 200, 2.8 ± 0.1 nm)	Perillaldehyde-isopropyl myristate/medium chain triglyceride	Hypolipidemic potential: decreased serum TC, TG, and LDL-C while increasing the HDL-C levels.	[111]
Liposomal nano-formulation (200 nm, spherical and homogenous, with no sign of coalescence)	Perillaldehyde from Perilla frutescens	Decrease in TC, TG, and LDL-C, increase in the HDL-C levels and the activities of SOD and GSH-Px.	[112]

CVDs: cardiovascular diseases; LDL-C: low-density lipoprotein cholesterol; HDL-C: high-density lipoprotein cholesterol; GSH-Px: glutathione peroxidase; SOD: superoxide dismutase.

## Abbreviations

ACC: CoA-carboxylase; Apo: apolipoprotein; BATs: brown adipose tissues; BMI: body mass index; CETP: cholesterol ester transfer protein; CVDs: cardiovascular diseases; FAS: fatty acid synthase; FFA: free fatty acids; FDA: Food and Drug Administration; IL: interleukin; HDL: high-density lipoprotein; GSH-Px: glutathione peroxidase; LDL: low-density lipoprotein; LDL-C: low-density lipoprotein cholesterol; mRNA: messenger ribonucleic acid; MDA: malondialdehyde; MCP-1: monocyte chemoattractant protein-1; MUO: metabolic unhealthy obesity; 1; MHO: metabolic healthy obesity; NO: nitric oxide; PCL: polycaprolactone; PEG: polyethylene glycol; PGMC: polymer-granular matter composite; PLGA-b: poly (lactic-co-glycolic acid)-b; PCL: polycaprolactone; PPAR: peroxisome proliferator-activated receptor 1; PVAT: perivascular adipose tissue; ROS: reactive oxygen species; T2DM: type-2 diabetes mellitus; TNF: tumor necrosis factor; TG: triglyceride; VLDL: very-low-density lipoprotein; SOD: superoxide dismutase; WATs: white adipose tissues; WHO: WC: World Health Organization; WC: waist circumference.

## References

[1] Sandoval-Vargas D., Concha-Rubio N.D., Navarrete P., Castro M., Medina D.A. (2021). Short Communication: Obesity Intervention Resulting in Significant Changes in the Human Gut Viral Composition. Appl. Sci..

[2] World Health Organization (2004). International Statistical Classification of Diseases and Related Health Problems: Alphabetical Index.

[3] Obesity and Overweight. https://www.who.int/news-room/fact-sheets/detail/obesity-and-overweight.

[4] Lobstein T., Hannah B., Neveux M. (2022). World Obesity Atlas.

[5] Trandafir L.M., Russu G., Moscalu M., Miron I., Lupu V.V., Leon Constantin M.M., Cojocaru E., Lupu A., Frasinariu O.E. (2020). Waist circumference a clinical criterion for prediction of cardio-vascular complications in children and adolescences with overweight and obesity. Medicine.

[6] Sioka C., Zotou P., Papafaklis M.I., Bechlioulis A., Sakellariou K., Rammos A., Gkika E., Lakkas L., Alexiou S., Kekiopoulos P. (2022). Body Mass Index Is Independently Associated with the Presence of Ischemia in Myocardial Perfusion Imaging. Medicina.

[7] Han T.S., Lean M.E. (2016). A clinical perspective of obesity, metabolic syndrome and cardiovascular disease. JRSM Cardiovasc. Dis..

[8] Al-Gindan Y.Y., Hankey C.R., Govan L., Gallagher D., Heymsfield S.B., Lean M.E. (2015). Derivation and validation of simple anthropometric equations to predict adipose tissue mass and total fat mass with MRI as the reference method. Br. J. Nutr..

[9] Schütz F., Figueiredo-Braga M., Barata P., Cruz-Martins N. (2021). Obesity and gut microbiome: Review of potential role of probiotics. Portoc. Biomed. J..

[10] Scudiero O., Pero R., Ranieri A., Terracciano D., Fimiani F., Cesaro A., Gentile L., Leggiero E., Laneri S., Moscarella E. (2019). Childhood obesity: An overview of laboratory medicine, exercise and microbiome. Clin. Chem. Lab. Med..

[11] Al-Assal K., Martinez A.C., Torrinhas S.R., Cardinelli C., Waitzberg D. (2018). Gut microbiota and obesity. Clin. Nutr. Exp..

[12] Engin A. (2017). The Definition and Prevalence of Obesity and Metabolic Syndrome. Adv. Exp. Med. Biol..

[13] Jakubiak G.K., Osadnik K., Lejawa M., Kasperczyk S., Osadnik T., Pawlas N. (2021). Oxidative Stress in Association with Metabolic Health and Obesity in Young Adults. Oxid. Med. Cell. Longev..

[14] Kruger H.S., De Lange-Loots Z., Kruger I.M., Pieters M. (2022). The Metabolic Profiles of Metabolically Healthy Obese and Metabolically Unhealthy Obese South African Adults over 10 Years. Int. J. Environ. Res. Public Health.

[15] Stanek A., Brozyna-Tkaczyk K., Myslinski W. (2021). The Role of Obesity-Induced Perivascular Adipose Tissue (PVAT) Dysfunction in Vascular Homeostasis. Nutrients.

[16] Bikel S., López-Leal G., Cornejo-Granados F., Gallardo-Becerra L., García-López R., Sánchez F., Equihua-Medina E., Ochoa-Romo J.P., López-Contreras B.E., Canizales-Quinteros S. (2021). Gut dsDNA virome shows diversity and richness alterations associated with childhood obesity and metabolic syndrome. iScience.

[17] Ash G.I., Dongin K., Mahua C. (2019). Promises of nanotherapeutics in obesity. Trends Endocrinol. Metab..

[18] Ealey K.N., Phillips J., Sung H.-K. (2021). COVID-19 and obesity: Fighting two pandemics with intermittent fasting. Trends Endocrinol. Metab..

[19] Dias S., Paredes S., Ribeiro L. (2018). Drugs involved in dyslipidemia and obesity treatment: Focus on adipose tissue. Int. J. Endocrinol..

[20] Hedayatnia M., Asadi Z., Zare-Feyzabadi R., Khorasani M.Y., Ghazizadeh H., Ghaffarian-Zirak R., Nosrati-Tirkani A., Mohammadi-Bajgiran M., Rohban M., Sadabadi F. (2020). Dyslipidemia and cardiovascular disease risk among the MASHAD study population. Lipids Health Dis..

[21] Su X., Daoquan P. (2020). The exchangeable apolipoproteins in lipid metabolism and obesity. Clin. Chim. Acta.

[22] Bays H.E., Toth P.P., Kris-Etherton P.M., Abate N., Aronne L.J., Brown W.V., Gonzalez-Campoy J.M., Jones S.R., Kumar R., La Forge R. (2013). Obesity, adiposity, and dyslipidemia: A consensus statement from the National Lipid Association. J. Clin. Lipidol..

[23] Skinner A.C., Perrin E.M., Moss L.A., Skelton J.A. (2015). Cardiometabolic Risks and Severity of Obesity in Children and Young Adults. N. Engl. J. Med..

[24] Klop B., Elte J.W., Cabezas M.C. (2013). Dyslipidemia in obesity: Mechanisms and potential targets. Nutrients.

[25] Feingold K.R., Feingold K.R., Anawalt B., Boyce A., Chrousos G., de Herder W.W., Dhatariya K., Dungan K., Hershman J.M., Hofland J., Kalra S. (2020). Obesity and Dyslipidemia.

[26] Zhang T., Chen J., Tang X., Luo Q., Xu D., Yu B. (2019). Interaction between adipocytes and high-density lipoprotein:new insights into the mechanism of obesity-induced dyslipidemia and atherosclerosis. Lipids Health Dis..

[27] Bjornson E., Adiels M., Taskinen M.R., Boren J. (2017). Kinetics of plasma triglycerides in abdominal obesity. Curr. Opin. Lipidol..

[28] Franssen R., Monajemi H., Stroes E.S., Kastelein J.J. (2011). Obesity and dyslipidemia. Med. Clin. N. Am..

[29] Jakubiak G.K., Cieślar G., Stanek A. (2022). Nitrotyrosine, Nitrated Lipoproteins, and Cardiovascular Dysfunction in Patients with Type 2 Diabetes: What Do We Know and What Remains to Be Explained?. Antioxidants.

[30] Lorey M.B., Öörni K., Kovanen P.T. (2022). Modified Lipoproteins Induce Arterial Wall Inflammation During Atherogenesis. Front. Cardiovasc. Med..

[31] Tsou Y.H., Wang B., Ho W., Hu B., Tang P., Sweet S., Zhang X.Q., Xu X. (2019). Nanotechnology-Mediated Drug Delivery for the Treatment of Obesity and Its Related Comorbidities. Adv. Health Mater..

[32] Velazquez A., Apovian C.M. (2018). Updates on obesity pharmacotherapy. Ann. N. Y. Acad. Sci..

[33] Joyce P., Meola T.R., Schultz H.B., Clive A. (2020). Prestidge, Biomaterials that regulate fat digestion for the treatment of obesity. Trends Food Sci. Technol..

[34] Vekic J., Zeljkovic A., Stefanovic A., Jelic-Ivanovic Z., Spasojevic-Kalimanovska V. (2019). Obesity and dyslipidemia. Metabolism.

[35] Sbraccia P., Finer N. (2019). Obesity: Pathogenesis, Diagnosis, and Treatment.

[36] Khan S., Mansoor S., Rafi Z., Kumari B., Shoaib A., Saeed M., Alshehri S., Ghoneim M.M., Rahamathulla M., Hani U. (2022). A review on nanotechnology: Properties, applications, and mechanistic insights of cellular uptake mechanisms. J. Mol. Liq..

[37] Durán-Lobato M., López-Estévez A.M., Cordeiro A.S., Dacoba T.G., Crecente-Campo J., Torres D., Alonso M.J. (2021). Nanotechnologies for the delivery of biologicals: Historical perspective and current landscape. Adv. Drug Deliv. Rev..

[38] Kebede M.A.I., Imae T., Ariga K., Aono M. (2019). Chapter 1.1—Low-Dimensional Nanomaterials. Micro and Nano Technologies, Advanced Supramolecular Nanoarchitectonics.

[39] Alshareeda A.T., Nur Khatijah M.Z., Al-Sowayan B.S. (2022). Nanotechnology: A revolutionary approach to prevent breast cancer recurrence. Asian J. Surg..

[40] Sandhu J.S., Das R., Mehta D.K., Dhanawat M. (2021). Virtue of Nanotechnology in Confronting Obesity: Recent Advances. Nanosci. Nanotechnol. Asia Bentham Sci. Publ..

[41] Wyatt H.R. (2013). Update on treatment strategies for obesity. J. Clin. Endocrinol. Metabol..

[42] Thovhogi N., Sibuyi N., Meyer M., Onani M., Madiehe A. (2015). Targeted delivery using peptide-functionalised gold nanoparticles to white adipose tissues of obese rats. J. Nanopart. Res..

[43] Hossen M.N., Kajimoto K., Akita H., Hyodo M., Harashima H. (2013). A comparative study between nanoparticle-targeted therapeutics and bioconjugates as obesity medication. J. Contr. Release.

[44] Hossen M.N., Kajimoto K., Akita H., Hyodo M., Ishitsuka T., Harashima H. (2013). Therapeutic assessment of cytochrome C for the prevention of obesity through endothelial cell-targeted nanoparticulate system. Mol. Ther..

[45] Hossen M.N., Kajimoto K., Akita H., Hyodo M., Harashima H. (2012). Vascular-targeted nanotherapy for obesity: Unexpected passive targeting mechanism to obese fat for the enhancement of active drug delivery. J. Control. Release.

[46] Hossen M.N., Kajimoto K., Akita H., Hyodo M., Ishitsuka T., Harashima H. (2010). Ligand-based targeted delivery of a peptide modified nanocarrier to endothelial cells in adipose tissue. J. Control. Release.

[47] Xue Y., Xu X., Zhang X.Q., Farokhzad O.C., Langer R. (2016). Preventing diet-induced obesity in mice by adipose tissue transformation and angiogenesis using targeted nanoparticles. Proc. Natl. Acad. Sci. USA.

[48] Sangwai M., Sardar S., Vavia P. (2014). Nanoemulsified orlistat-embedded multi-unit pellet system (MUPS) with improved dissolution and pancreatic lipase inhibition. Pharma Dev. Technol..

[49] Chen Y.L., Zhu S., Zhang L., Feng P.J., Yao X.K., Qian C.G., Zhang C., Jiang X.Q., Shen Q.D. (2016). Smart conjugated polymer nanocarrier for healthy weight loss by negative feedback regulation of lipase activity. Nanoscale.

[50] Marrache S., Dhar S. (2012). Engineering of blended nanoparticle platform for delivery of mitochondria-acting therapeutics. Proc. Natl. Acad. Sci. USA.

[51] Jiang C., Cano-Vega M.A., Yue F., Kuang L., Narayanan N., Uzunalli G., Merkel M.P., Kuang S., Deng M. (2017). Dibenzazepine-Loaded Nanoparticles Induce Local Browning of White Adipose Tissue to Counteract Obesity. Mol. Ther..

[52] Ma L., Liu T.W., Wallig M.A., Dobrucki I.T., Dobrucki L.W., Nelson E.R., Swanson K.S., Smith A.M. (2016). Efficient Targeting of Adipose Tissue Macrophages in Obesity with Polysaccharide Nanocarriers. ACS Nano..

[53] Di Mascolo D., Lyon C.J., Aryal S., Ramirez M.R., Wang J., Candeloro P., Guindani M., Hsueh W.A., Decuzzi P. (2013). Rosiglitazone-loaded nanospheres for modulating macrophage-specific inflammation in obesity. J. Control. Release.

[54] Zhang Y., Yu J., Qiang L., Gu Z. (2018). Nanomedicine for obesity treatment. Sci. China Life Sci..

[55] Sibuyi N.R.S., Moabelo K.L., Meyer M., Onani M.O., Dube A., Madiehe A.M. (2019). Nanotechnology advances towards development of targeted-treatment for obesity. J. Nanobiotechnol..

[56] Sharifi S., Daghighi S., Motazacker M., Badlou B.A., Sanjabi B., Akbarkhanzadeh A., Rowshani A.T., Laurent S., Peppelenbosch M.P., Rezaee F. (2013). Superparamagnetic iron oxide nanoparticles alter expression of obesity and T2D-associated risk genes in human adipocytes. Sci. Rep..

[57] Rocca A., Moscato S., Ronca F., Nitti S., Mattoli V., Giorgi M., Ciofani G. (2015). Pilot in vivo investigation of cerium oxide nanoparticles as a novel anti-obesity pharmaceutical formulation. Nanomedicine.

[58] Zhang H.L., Zhong X.B., Tao Y., Wu S.H., Su Z.Q. (2012). Effects of chitosan and water-soluble chitosan micro- and nanoparticles in obese rats fed a high-fat diet. Int. J. Nanomed..

[59] Kupferschmidt N., Csikasz R.I., Ballell L., Bengtsson T., Garcia-Bennett A.E. (2014). Large pore mesoporous silica induced weight loss in obese mice. Nanomedicine.

[60] Sheng W., Alhasan A.H., DiBernardo G., Almutairi K.M., Rubin J.P., DiBernardo B.E., Almutairi A. (2015). Gold Nanoparticle-assisted Selective Photothermolysis of Adipose Tissue (NanoLipo). Plast. Reconstr. Surg. Glob. Open.

[61] Lee J.H., Jeong H.S., Lee D.H., Beack S., Kim T., Lee G.H., Park W.C., Kim C., Kim K.S., Hahn S.K. (2017). Targeted Hyaluronate-Hollow Gold Nanosphere Conjugate for Anti-Obesity Photothermal Lipolysis. ACS Biomater. Sci. Eng..

[62] Chen H., Ng J.P.M., Tan Y., McGrath K., Bishop D.P., Oliver B., Chan Y.L., Cortie M.B., Milthorpe B.K., Valenzuela S.M. (2018). Gold nanoparticles improve metabolic profile of mice fed a high-fat diet. J. Nanobiotechnol..

[63] Ipsen D.H., Tveden-Nyborg P., Lykkesfeldt J. (2016). Dyslipidemia: Obese or Not Obese-That Is Not the Question. Curr. Obes. Rep..

[64] Han Z., Wang Y., Li J. (2021). Effects of Atorvastatin Combined with Nano-Selenium on Blood Lipids and Oxidative Stress in Atherosclerotic Rats. J. Nanosci. Nanotechnol..

[65] Farrag S.M., Hamzawy M.A., El-Yamany M.F., Saad M.A., Nassar N.N. (2018). Atorvastatin in nano-particulate formulation abates muscle and liver affliction when coalesced with coenzyme Q10 and/or vitamin E in hyperlipidemic rats. Life Sci..

[66] Mathur M., Devi Vemula K. (2018). Investigation of different types of nano drug delivery systems of atorvastatin for the treatment of hyperlipidemia. Drug Dev. Ind. Pharm..

[67] Harisa G.I., Alomrani A.H., Badran M.M. (2017). Simvastatin-loaded nanostructured lipid carriers attenuate the atherogenic risk of erythrocytes in hyperlipidemic rats. Eur. J. Pharm. Sci..

[68] Rizvi S.Z.H., Shah F.A., Khan N., Muhammad I., Ali K.H., Ansari M.M., Din F.U., Qureshi O.S., Kim K.W., Choe Y.H. (2019). Simvastatin-loaded solid lipid nanoparticles for enhanced anti-hyperlipidemic activity in hyperlipidemia animal model. Int. J. Pharm..

[69] Ahsan M.N., Prasad Verma P.R. (2017). Solidified self nano-emulsifying drug delivery system of rosuvastatin calcium to treat diet-induced hyperlipidemia in rat: In vitro and in vivo evaluations. Ther. Deliv..

[70] Momtazi-Borojeni A.A., Abdollahi E., Jaafari M.R., Banach M., Watts G.F., Sahebkar A. (2022). Negatively-charged Liposome Nanoparticles Can Prevent Dyslipidemia and Atherosclerosis Progression in the Rabbit Model. Curr. Vasc. Pharmacol..

[71] He H., Ghosh S., Yang H. (2017). Nanomedicines for dysfunctional macrophage-associated diseases. J. Control. Release.

[72] Hafez D.A., Elkhodairy K.A., Teleb M., Elzoghby A.O. (2020). Nanomedicine-based approaches for improved delivery of phyto-therapeutics for cancer therapy. Expert Opin. Drug Deliv..

[73] Wachtel-Galor S., Benzie I.F.F., Benzie I.F.F., Wachtel-Galor S. (2011). Herbal Medicine: An Introduction to Its History, Usage, Regulation, Current Trends, and Research Needs. Herbal Medicine: Biomolecular and Clinical Aspects.

[74] Karri S., Sharma S., Hatware K., Patil K. (2019). Natural anti-obesity agents and their therapeutic role in management of obesity: A future trend perspective. Biomed. Pharmacother..

[75] Hasani-Ranjbar S., Nayebi N., Moradi L., Mehri A., Larijani B., Abdollahi M. (2010). The efficacy and safety of herbal medicines used in the treatment of hyperlipidemia; a systematic review. Curr. Pharm. Des..

[76] Lenon G.B., Li K.X., Chang Y.H., Yang A.W., Da Costa C., Li C.G., Cohen M., Mann N., Xue C.C. (2012). Efficacy and Safety of a Chinese Herbal Medicine Formula (RCM-104) in the Management of Simple Obesity: A Randomized, Placebo-Controlled Clinical Trial. Evid. Based Complement. Altern. Med..

[77] Hioki C., Yoshimoto K., Yoshida T. (2004). Efficacy of bofu-tsusho-san, an oriental herbal medicine, in obese Japanese women with impaired glucose tolerance. Clin. Exp. Pharmacol. Physiol..

[78] Bahmani M., Eftekhari Z., Saki K., Fazeli-Moghadam E., Jelodari M., Rafieian-Kopaei M. (2016). Obesity Phytotherapy: Review of Native Herbs Used in Traditional Medicine for Obesity. J. Evid. Based Complement. Altern. Med..

[79] Eldalo A.S., Alotaibi M.N., Alenazi T.O., Albogami H.A., Mohamed K.M. (2017). Use of Herbal Medicines in the Treatment of Obesity in Taif, Saudi Arabia. Saudi J. Med. Med. Sci..

[80] Liu Y., Sun M., Yao H., Liu Y., Gao R. (2017). Herbal Medicine for the Treatment of Obesity: An Overview of Scientific Evidence from 2007 to 2017. Evid. Based Complement. Alternat. Med..

[81] Shende P., Narvenker R. (2021). Herbal nanotherapy: A new paradigm over conventional obesity treatment. J. Drug Deliv. Sci. Technol..

[82] Fang H., Dong-Sheng S., Kai-Li W., Dan-Ying S. (2022). Nanomedicine of Plant Origin for the Treatment of Metabolic Disorders. Front. Bioeng. Biotechnol..

[83] Gao L., Hu Y., Hu D., Li Y., Yang S., Dong X., Alharbi S.A., Liu H. (2020). Anti-obesity Activity of Gold Nanoparticles Synthesized from Salacia Chinensis Modulates the Biochemical Alterations in High-Fat Diet-Induced Obese Rat Model via AMPK Signaling Pathway. Arabian J. Chem..

[84] Ansari S., Bari A., Ullah R., Mathanmohun M., Veeraraghavan V.P., Sun Z. (2019). Gold Nanoparticles Synthesized with Smilax Glabra Rhizome Modulates the Anti-obesity Parameters in High-Fat Diet and Streptozotocin Induced Obese Diabetes Rat Model. J. Photochem. Photobiol. B Biol..

[85] Li W., Wan H., Yan S., Yan Z., Chen Y., Guo P., Ramesh T., Cui Y., Ning L. (2020). Gold nanoparticles synthesized with Poria cocos modulates the anti-obesity parameters in high-fat diet and streptozotocin induced obese diabetes rat model. Arab. J. Chem..

[86] Saratale G.D., Saratale R.G., Benelli G., Kumar G., Pugazhendhi A., Kim D.-S., Shin H.-S. (2017). Anti-diabetic Potential of Silver Nanoparticles Synthesized with Argyreia Nervosa Leaf Extract High Synergistic Antibacterial Activity with Standard Antibiotics against Foodborne Bacteria. J. Clust. Sci..

[87] Zu Y., Zhao L., Hao L., Mechref Y., Zabet-Moghaddam M., Keyel P.A., Abbasi M., Wu D., Dawson J.A., Zhang R. (2021). Browning white Adipose Tissue Using Adipose Stromal Cell-Targeted Resveratrol-Loaded Nanoparticles for Combating Obesity. J. Control. Release.

[88] Ariamoghaddam A.R., Ebrahimi-Hosseinzadeh B., Hatamian-Zarmi A., Sahraeian R. (2018). In Vivo anti-obesity Efficacy of Curcumin Loaded Nanofibers Transdermal Patches in High-Fat Diet Induced Obese Rats. Mater. Sci. Eng. C.

[89] El-Menshawe S., Ali A., Rabeh M., Khalil N. (2018). Nanosized Soy Phytosome-Based Thermogel as Topical Anti-obesity Formulation: An Approach for Acceptable Level of Evidence of an Effective Novel Herbal Weight Loss Product. Int. J. Nanomed..

[90] Lee M.-S., Jung S., Shin Y., Lee S., Kim C.-T., Kim I.-H., Kim Y. (2017). Lipolytic Efficacy of Alginate Double-Layer Nanoemulsion Containing Oleoresin Capsicum in Differentiated 3T3-L1 Adipocytes. Food Nutr. Res..

[91] Berger E., Colosetti P., Jalabert A., Meugnier E., Wiklander O.P.B., Jouhet J., Errazurig-Cerda E., Chanon S., Gupta D., Rautureau G. (2020). Use of Nanovesicles from Orange Juice to Reverse Diet-Induced Gut Modifications in Diet-Induced Obese Mice. Mol. Ther. Methods Clin. Dev..

[92] Yi M.H., Simu S.Y., Ahn S., Aceituno V.C., Wang C., Mathiyalagan R., Hurh J., Batjikh I., Ali H., Kim Y.-J. (2020). Anti-obesity Effect of Gold Nanoparticles fromDendropanax Morbifera Léveilleby Suppression of Triglyceride Synthesis and Downregulation of PPARγ and CEBPα Signaling Pathways in 3T3-L1 Mature Adipocytes and HepG2 Cells. Curr. Nanosci..

[93] Lacatusu I., Badea N., Udeanu D., Coc L., Pop A., Negut C.C., Tanase C., Stan R., Meghea A. (2019). Improved anti-obesity effect of herbal active and endogenous lipids co-loaded lipid nanocarriers: Preparation, in vitro and in vivo evaluation. Mater. Sci. Eng. C.

[94] Zu Y., Overby H., Ren G., Fan Z., Zhao L., Wang S. (2018). Resveratrol liposomes and lipid nanocarriers: Comparison of characteristics and inducing browning of white adipocytes. Colloids Surf. B Biointerfaces..

[95] Chen C.-H., Chen C.J., Elzoghby A.O., Yeh T.S., Fang J.Y. (2018). Self-assembly and directed assembly of lipid nanocarriers for prevention of liver fibrosis in obese rats: A comparison with the therapy of bariatric surgery. Nanomedicine.

[96] Ezhilarasi P., Muthukumar S., Anandharamakrishnan C. (2016). Solid lipid nanoparticle enhances bioavailability of hydroxycitric acid compared to a microparticle delivery system. RSC Adv..

[97] Nallamuthu I., Devi A., Khanum F. (2015). Chlorogenic acid loaded chitosan nanoparticles with sustained release property, retained antioxidant activity and enhanced bioavailability. Asian J. Pharm. Sci..

[98] Lee J.H., Kim J.-C. (2015). Effect of cubic phase nanoparticle on obesity-suppressing efficacy of herbal extracts. Biotechnol. Bioproc. Eng..

[99] Kozuka C., Shimizu-Okabe C., Takayama C., Nakano K., Morinaga H., Kinjo A., Fukuda K., Kamei A., Yasuoka A., Kondo T. (2017). Marked augmentation of PLGA nanoparticle-induced metabolically beneficial impact of γ-oryzanol on fuel dyshomeostasis in genetically obese-diabetic ob/ob mice. Drug Deliv..

[100] Sun Y.-E., Wang W., Qin J. (2018). Anti-hyperlipidemia of Garlic by Reducing the Level of Total Cholesterol and Low-Density Lipoprotein. Medicine.

[101] Khutami C., Sumiwi S.A., Khairul Ikram N.K., Muchtaridi M. (2022). The Effects of Antioxidants from Natural Products on Obesity, Dyslipidemia, Diabetes and Their Molecular Signaling Mechanism. Int. J. Mol. Sci..

[102] Toth P.P., Patti A.M., Nikolic D., Giglio R.V., Castellino G., Biancucci T., Geraci F., David S., Montalto G., Rizvi A. (2016). Bergamot Reduces Plasma Lipids, Atherogenic Small Dense LDL, and Subclinical Atherosclerosis in Subjects with Moderate Hypercholesterolemia: A 6 Months Prospective Study. Front. Pharmacol..

[103] Moohebati M., Yazdandoust S., Sahebkar A., Mazidi M., Sharghi-Shahri Z., Ferns G., Ghayour-Mobarhan M. (2014). Investigation of the effect of short-term supplementation with curcuminoids on circulating small dense low-density lipoprotein concentrations in obese dyslipidemic subjects: A randomized double-blind placebo-controlled cross-over trial. ARYA Atheroscler..

[104] Yang C., Xia H., Wan M., Lu Y., Xu D., Yang X., Yang L., Sun G. (2021). Comparisons of the effects of different flaxseed products consumption on lipid profiles, inflammatory cytokines and anthropometric indices in patients with dyslipidemia related diseases: Systematic review and a dose-response meta-analysis of randomized controlled trials. Nutr. Metab..

[105] Ragavan G., Muralidaran Y., Sridharan B., Nachiappa Ganesh R., Viswanathan P. (2017). Evaluation of Garlic Oil in Nano-Emulsified Form: Optimization and its Efficacy in High-Fat Diet Induced Dyslipidemia in Wistar Rats. Food Chem. Toxicol..

[106] Javanshir R., Honarmand M., Hosseini M., Hemmati M. (2020). Anti-dyslipidemic Properties of green Gold Nanoparticle: Improvement in Oxidative Antioxidative Balance and Associated Atherogenicity and Insulin Resistance. Clin. Phytosci..

[107] Cheong A.M., Jessica Koh J.X., Patrick N.O., Tan C.P., Nyam K.L. (2018). Hypocholesterolemic Effects of Kenaf Seed Oil, Macroemulsion, and Nanoemulsion in High-Cholesterol Diet Induced Rats. J. Food Sci..

[108] Al-Kurdy M.J., Khadim Khudair K. (2020). The Effect of Black Currant Selenium Nanoparticles on Dyslipidemia and Oxidant-Antioxidant Status in D-Galactose Treated Rats. Kufa J. Vet. Med. Sci..

[109] Ali Z.S., Khudair K.K. (2019). Synthesis, Characterization of Silver Nanoparticles Using Nigella Sativa Seeds and Study Their Effects on the Serum Lipid Profile and DNA Damage on the Rats’ Blood Treated with Hydrogen Peroxide. Iraqi J. Vet. Med..

[110] Al-Okbi S.Y., Hussein A.M.S., Elbakry H.F.H., Fouda K.A., Mahmoud K.F., Hassan M.E. (2018). Health Benefits of Fennel, Rosemary Volatile Oils and their Nano-Forms in Dyslipidemic Rat Model. Pak. J. Biol. Sci..

[111] Omari-Siaw E., Zhu Y., Wang H., Peng W., Firempong C.K., Wang Y.W., Cao X., Deng W., Yu J., Xu X. (2016). Hypolipidemic potential of perillaldehyde-loaded self-nanoemulsifying delivery system in high-fat diet induced hyperlipidemic mice: Formulation, in vitro and in vivo evaluation. Eur. J. Pharm. Sci..

[112] Omari-Siaw E., Wang Q., Sun C., Gu Z., Zhu Y., Cao X., Firempong C.K., Agyare R., Xu X., Yu J. (2016). Tissue distribution and enhanced in vivo anti-hyperlipidemic-antioxidant effects of perillaldehyde-loaded liposomal nanoformulation against Poloxamer 407-induced hyperlipidemia. Int. J. Pharm..

